# Genetic Testing in the Diagnosis of Primary Ciliary Dyskinesia: State-of-the-Art and Future Perspectives

**DOI:** 10.3390/jcm3020491

**Published:** 2014-05-09

**Authors:** Samuel A. Collins, Woolf T. Walker, Jane S. Lucas

**Affiliations:** 1 Primary Ciliary Dyskinesia Centre, University Hospital Southampton NHS Foundation Trust, Southampton SO16 6YD, UK; E-Mails: samacollins@gmail.com (S.A.C.); woolf.walker@uhs.nhs.uk (W.T.W.); 2 NIHR Southampton Respiratory Biomedical Research Unit, University of Southampton and University Hospital Southampton NHS Foundation Trust, Southampton SO16 6YD, UK; 3 Clinical and Experimental Sciences Academic Unit (Mail Point 803), University of Southampton Faculty of Medicine and University Hospital Southampton NHS Foundation Trust, Southampton SO16 6YD, UK

**Keywords:** primary ciliary dyskinesia, cystic fibrosis, genetic testing, screening, mutation, cilia, diagnosis

## Abstract

Primary ciliary dyskinesia (PCD) is a heterogeneous autosomal recessive condition affecting around 1:15,000. In people with PCD, microscopic motile cilia do not move normally resulting in impaired clearance of mucus and debris leading to repeated sinopulmonary infection. If diagnosis is delayed, permanent bronchiectasis and deterioration of lung function occurs. Other complications associated with PCD include congenital heart disease, hearing impairment and infertility. A small number of longitudinal studies suggest that lung function deteriorates before diagnosis of PCD but may stabilise following diagnosis with subsequent specialist management. Early diagnosis is therefore essential, but for a number of reasons referral for diagnostic testing is often delayed until older childhood or even adulthood. Functional diagnostic tests for PCD are expensive, time consuming and require specialist equipment and scientists. In the last few years, there have been considerable developments to identify genes associated with PCD, currently enabling 65% of patients to be identified by bi-allelic mutations. The rapid identification of new genes continues. This review will consider the evidence that early diagnosis of PCD is beneficial. It will review the recent advances in identification of PCD-associated genes and will discuss the role of genetic testing in PCD. It will then consider whether screening for PCD antenatally or in the new born is likely to become a feasible and acceptable for this rare disease.

## 1. Introduction

Primary ciliary dyskinesia (PCD) is an inherited disorder of the function of motile cilia and sperm flagella, usually associated with abnormalities of the cilial ultrastructure as observed by electron microscopy (EM). It has an incidence of around 1 in 15,000 live births [1,2], however it is considerably more prevalent in certain populations, for example a consanguineous British Asian population has a prevalence of 1:2265 [3]. The majority of patients are symptomatic from birth and go on to have persistent or recurrent sinopulmonary infection. Around half of patients have associated situs inversus (Kartagener’s syndrome) or other disorders of left-right asymmetry [4]. Diagnosis is often delayed with only half of cases identified before 5 years of age [2]. Evidence that later diagnosis is associated with poorer lung function and quality of life [5,6] highlights the need for early diagnosis. Cystic fibrosis (CF) shares a number of features with PCD including progressive bronchiectatic disease and decline in lung function. The advent of neonatal screening for CF has allowed earlier diagnosis with potential improvements in long-term lung function and morbidity [7].

Siewert first described the triad of situs inversus, bronchiectasis and sinusitis in 1904, with the term Kartagener’s syndrome coined following his 1935 description. In 1976, Afzelius was the first to link this disease to cilia and termed the disease “immotile cilia syndrome” [8]. *DNAH5* was identified as a candidate gene for PCD in 2000 [9] and shortly after was characterized and confirmed to be associated with PCD and randomization of left-right symmetry [10]. Rapid advances in understanding the molecular genetic basis of PCD have been made in recent years.

**Figure 1:**
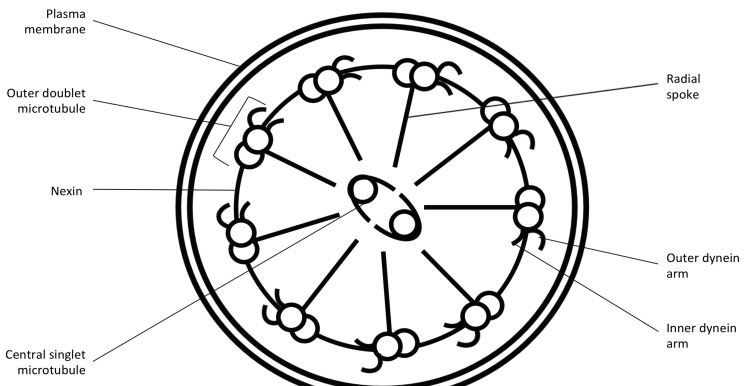
Diagram showing the main structural elements of the human motile cilium with the “9 + 2” arrangement.

**Figure 2:**
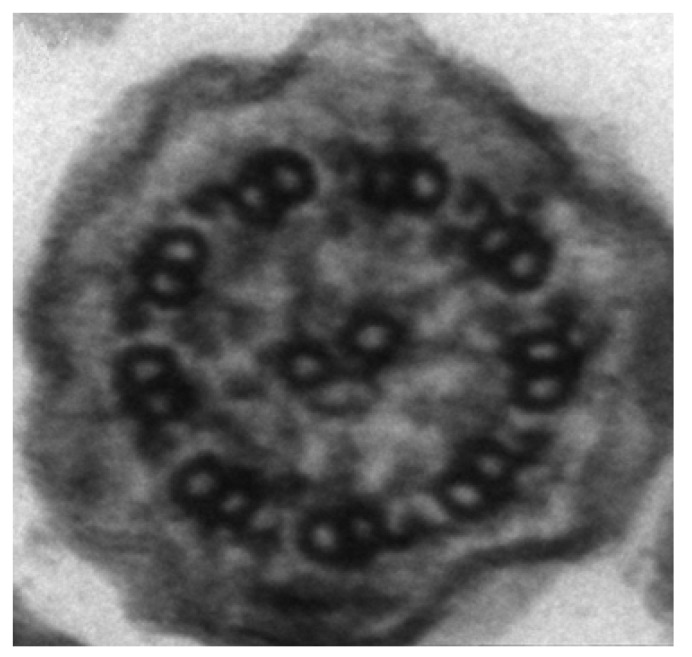
Scanning electron microscope image of a human cilium with normal ultrastructure.

Motile cilia are found in respiratory epithelium, brain ependymal cells, spinal cord and fallopian tubes whilst sharing a common axonemal structure with spermatozoa flagella. Cilial axonemes of healthy individuals have a “9 + 2” arrangement with nine pairs of microtubule doublets surrounding a central pair running the length of the ciliary axoneme (Figure 1 and Figure 2). Attached to the peripheral microtubules are inner and outer dynein arms in which dynein, a mechanochemical ATPase, generates the force for ciliary beating. The organized structure is maintained by nexin and radial spokes. Defects in the ultrastructure cause the cilia to beat abnormally, impairing mucociliary clearance leading to sinopulmonary disease, glue ear and female sub-fertility. Abnormalities of the ultrastructure of sperm flagella can lead to dysmotility and infertility. PCD in humans rarely causes hydrocephalus. The “9 + 0” motile cilia (no central pair) are present on the embryonic ventral node and have a role in determining left-right symmetry. Abnormal ciliary function in these embryonal cilia can lead to the classic situs inversus described by Kartagener where the organs are a mirror image or other disorders of situs termed heterotaxy (including left isomerism, right isomerism, isolated dextrocardia and abdominal situs inversus) [4,11].

Consistent with the many proteins involved in ciliary structure, protein assembly and intraflagellar transport PCD is a markedly heterogeneous disorder. To date, almost 30 genes have been found to be associated with PCD [12], mostly linked to specific ultrastructural defects, but it is anticipated that several hundred genes may code for proteins responsible for normal ciliary function.

Expanding knowledge of PCD genetics has raised the possibility of genetic screening for the condition, similarly to neonatal CF screening programs. Approximately 50%–60% of PCD patients have bi-allelic mutations in genes that are currently known to be associated with PCD making testing to support diagnosis a reality [12]. Current hurdles include the large number of PCD genes that are as yet unidentified, lack of a good supporting screening test for neonates, and the relatively low incidence of the disease in most populations. This manuscript reviews the need for, and possibility of, early screening for PCD in high-risk populations.

## 2. Diagnosis of PCD

Diagnostic investigations for PCD are highly specialised, requiring expensive equipment and an experienced team of clinicians and scientists [13]. European consensus guidelines [14] reflect the need for access to a number of methods to ensure robust diagnosis, as there is no single “gold standard” investigation.

Ciliary defects in PCD were first identified using electron microscopy [8] and this remained the “gold standard” diagnostic tool for many years. Samples of ciliated respiratory epithelium can be collected from nasal or bronchial brushing biopsy and approximately 100 cilia examined in transverse section to identify abnormalities of the outer or inner dynein arms, central pairs or microtubular arrangement [15]. A 3%–30% of patients with PCD are reported to have normal ultrastructure [16,17] and assessment of ciliary function is therefore necessary to exclude PCD. Ciliated respiratory cells obtained by brush biopsy can be imaged by high resolution, high-speed video (HSV) microscopy [18]. The images are played back at slower speed to allow analysis of beat pattern. Ciliary beat frequency is normally 11–18 Hz (measured at 37 °C) but in patients with PCD the beat frequency is typically static, slow or hyperfrequent. Less often the beat frequency is normal but the sweep or beat pattern abnormal [15]. These dysmotile patterns are often associated with specific transmission electron microscope (TEM) defects [19], for example in outer dynein arm (ODA) or combined inner dynein arm (IDA) and ODA defects, the majority of cilia are static, whilst with central pair defects cilia make a rotating motion rather than sweeping. Recently, subtle abnormalities of beat pattern [20] have been shown to be associated with the full PCD phenotype demonstrating the need for analysis of ciliary beat pattern by experienced technicians. HSV analysis is frequently complicated by secondary ciliary dyskinesia, which is common in patients with viral infections or simply due to damage to the epithelium during sampling [21].

In recent years, additional diagnostic tests have been added to support the diagnostic portfolio, particularly where HSV analysis and electron microscope (EM) examination are inconclusive. Cell culture of biopsy samples prior to re-analysis by HSV and EM minimises secondary ciliary defects [22] and immunofluorescent analysis of proteins can define ciliary defects [23]. Radioaerosol mucociliary clearance provides an *in vivo* assessment of ciliary function [24]. However, these additional investigations are only available at a handful of centres, often as a research rather than clinical tool. Genetic testing is not yet routine in most countries but the recent rapid increase in identified mutations that is reviewed below makes it likely that PCD genetics testing will soon become part of the diagnostic pathway.

Nasal nitric oxide (nNO) is extremely low in most, but not all, patients with PCD and therefore provides a good screening test [25,26,27,28]. Nasal NO can be measured using commercially available analysers, which sample gas from the upper airway (transnasal flow) during breath-holding or tidal breathing [29]. Infants with PCD have low nNO [30,31], and measurement during tidal breathing is possible in most young children [32]. The main drawback of nNO as a screening test in infants is poor specificity since approximately 40% of healthy young children have low levels [33]. There is therefore no reliable screening test in the group who would benefit most from screening.

In summary, ciliary EM and HSV are technically challenging, labour intensive processes that should be undertaken only in specialist referral centres. Expanded genetic testing might help in identifying patients that need functional assessment and improving the diagnostic process, thus impacting on the need to diagnose patients at an early age.

## 3. The Need for Early Diagnostics

The majority of PCD patients have neonatal symptoms and around half have situs inversus but, despite these signs, diagnosis is often delayed. This prevents early onset of regular airway clearance therapy, aggressive management of infections, monitoring and treatment of hearing impairment and genetic counselling for the family. In a series of 55 cases, Coren *et al.* found the median age at diagnosis to be 4 years, even though 67% had neonatal respiratory distress and 69% had abnormal situs [5] (with a prevalence of 50% in PCD, this suggests under-recognition in those with normal situs). Although 45% of patients had both neonatal symptoms and situs inversus, only half of these children were diagnosed before the age of 1 year [5]. Recent survey data from across Europe [2] showed the average age at diagnosis to be 5.8 years in those without situs inversus and 3.5 years in those with it. It was also noted that diagnosis was earlier in centres caring for more than 20 PCD patients (3 years *vs.* 4 years) emphasizing the importance of clinical suspicion and access to diagnostic tests [2]. By comparison, the average age of diagnosis for CF is 1.3 years even though it is unusual for these patients to have any neonatal symptoms unless they present with meconium ileus [5]. This emphasises the importance of clinical suspicion and identification of those at risk as any advances in genetic testing will only be useful once applied to the appropriate group of patients.

Early diagnosis of PCD has the potential to improve patient outcomes; 12 of the patients in the Coren series already had bronchiectasis at diagnosis [5]. Age at diagnosis has been shown to affect long-term lung function [6] whilst an observational study from North America [34] highlighted that PCD can lead to severe respiratory disease in adulthood with a high percentage developing respiratory failure and requiring lung transplant. Ellerman and Bisgaard showed that, unlike CF, lung function was relatively stable once therapy with antibiotics and airway clearance was initiated [6]. It is anticipated that whilst early diagnosis will delay disease progression and improve morbidity, knowledge of the diagnosis can also direct appropriate therapy choice; for example, treatment of hearing impairment and rhinosinusitis should be treated by specialists with an understanding of PCD as treatment options may be different to the general population.

PCD is therefore a disease that presents with early symptoms and can progress to significant, irreversible lung disease but which is amenable to early intervention meaning there are significant potential benefits from early diagnosis.

## 4. Genetics of PCD

PCD is primarily an autosomal recessive disease. Unlike CF, PCD is a markedly genetically heterogeneous condition with mutations in the 27 known genes (Table 1) accounting for 50%–60% of PCD cases [12]. Whilst the *CFTR* gene associated with CF was identified in 1989 [35], it was not until 2000 that the first gene associated with PCD was reported [9]. The gene was *DNAH5* which is a cause of defects of the dynein arms. *DNAI1* identification followed soon after [36]. *DNAI1* or *DNAH5* mutations account for the majority of genetic mutations in North America [10,37]. Approximately 50%–60% of PCD patients have bi-allelic mutations in a known PCD gene. Most of these mutations correspond to a specific ultrastructural defect. For example, *ZMYND10* [38] and *DYX1C1* [39] are associated with inner and outer dynein arm defects whilst mutations in *CCDC39* and *CCDC40* lead to axonemal disorganisation and absent inner dynein arms [40]. *DNAH11* was identified in 2002 in a patient with normal ciliary ultrastructure on EM [41] and accounts for 22% of those with normal ultrastructure [42]. Recently, mutations of the *HYDIN* gene were noted to be associated with apparently normal ultrastructure using conventional EM but by using a tomography approach an abnormality of the central pair apparatus was seen [20]. Many of the early identifications of genes used a candidate gene approach but recent discoveries such as *HEATR2* and *ARMC4* have been made through ciliome, exome or whole genome sequencing [43,44].

**Table 1:** Genes with mutations linked to primary ciliary dyskinesia. ODA—outer dynein arms, IDA—inner dynein arms.

Gene	Structural Defect
**Abnormalities in dynein proteins**	
*DNAI1*	ODA defect (+/− IDA)
*DNAH5*	ODA defect (+/− IDA)
*DNAH11*	Beat abnormalities (normal structure)
*DNAI2*	ODA defect
*DNALI1*	ODA defect
*TXNDC3*	ODA defect
*ARMC4*	ODA defect
**Genes coding for proteins responsible for assembly or transport of axonemal proteins**	
*KTU*	ODA and IDA defects
*LRRC50*	ODA and IDA defects
*DNAAF3*	ODA and IDA defects
*CCDC39*	ODA and IDA defects
*CCDC40*	Axone disorganisation and IDA defect
*CCDC103*	ODA and IDA defects
*CCDC114*	ODA defect
*HEATR2*	Absent ODA
*CCDC65*	Cilial vibration, normal structure
*ZMYND10*	Absent ODA + IDA
*SPAG1*	Absent ODA + IDA
*C21orf59*	Absent ODA + IDA
**Central pair abnormalities**	
*RSPH9*	Central pair defects
*RSPH4A*	Central pair defects
*RSPH1*	Central pair defects
*HYDIN*	Central pair defects
**Nexin-dynein complex defects**	
*DRC CCDC164*	Nexin link missing
*CCDC65*	Beat abnormalities
**Genes causing PCD with associated syndromes**	
*OFD1*	Unknown
*RPGR*	Variable

The majority of PCD associated genes are rare and sometimes linked to only one or two affected families. Genetic diagnostics might therefore focus on a few of the more common mutations, much as CF screening programs concentrate on only a few of the almost 2000 known *CFTR* mutations [45].

Patients with identified mutations generally have biallelic mutations at a single locus, for example two mutated copies of *DNAH5*. Given the number of different possible mutations at each locus, the disease phenotype will often be the result of compound heterozygosity with the potential of mutations at different loci combining to cause a clinical phenotype. Indeed, single allele *DNAH5* mutations are found in around 7% of PCD patients with no other mutation found [37]. Similarly, a patient with a single allele *DNAH11* mutation has been reported with a classical PCD phenotype [46].

A challenge facing researchers seeking new mutations is the huge volume of genetic data generated by advances in sequencing and the need for sophisticated bioinformatics. Techniques such as whole exome sequencing produce a huge number of variations and require sophisticated algorithms to analyse and process these variations. International collaborations advancing our knowledge of disease associated variation ensure data can be appropriately analysed and verified [47].

## 5. Comparison of Primary Ciliary Dyskinesia and Cystic Fibrosis

Although PCD and CF are both recessively inherited causes of chronic suppurative lung disease, there are a number of differences in both pathogenesis and aetiology. CF is associated with defects of a single gene, the cystic fibrosis transmembrane conductance regulator (*CFTR*) gene which encodes the chloride channel of the same name. To date, almost 2000 *CFTR* mutations have been identified [45]. Targeted mutation analysis with the American College of Medical Genetics 25 mutation panel detects at least one mutation in 88% of CF cases [48] whilst sequence analysis detects up to 98.7% of known *CFTR* mutations [49]. Neonatal CF screening is now in place in a number of countries worldwide including the U.S., UK and Australia, and many parts of Europe, Russia and Canada; though not all of these include DNA analysis within their newborn screening program. All screening protocols rely on the collection of blood spots in the newborn period for analysis of immunoreactive trypsin (IRT) levels. In the UK, those with IRT above the 99.5th centile are sent for analysis of the four commonest mutations with two mutations in a patient resulting in a label of “probably CF”. The presence of a single mutation leads to further 29–31 gene analysis whilst the presence of no mutations leads to further IRT analysis [50,51]. The U.S. approach varies across states whilst European practice varies widely, for example, Poland use an initial 640 mutation panel with complete sequencing of the *CFTR* gene if only one mutation is found [52]. Although very sensitive, this last approach can yield a high number of mutations of variable penetrance and expression. The potential benefits of NBS in PCD were demonstrated by a study of CF in London that NBS showed a reduction in the median age at diagnosis (excluding those with meconium ileus) from 2.4 years to 3 weeks [53].

PCD is currently associated with 27 different genes but with several hundred different proteins potentially affected, the task of identifying disease causing mutations is made all the more difficult. This difficulty is reflected in the fact that only 60% of North American PCD cases have an identified genetic mutation [54], the same proportion that are accounted for by a single mutation in CF (δF508 homozygotes) [50]. Targeted mutation analysis has not been effective in PCD as each gene may have many different mutations associated with clinical disease, each of which is very rare; however next generation sequencing panels have been developed that can screen known genes by comparing them to reference genomes. Next generation sequencing techniques, such as whole exome sequencing, may also expand the number of known disease associated loci [43].

Another major difference in CF is that the NBS has IRT as a reliable investigation to screen patients prior to genetic testing [55]. Effective PCD screening should also rely on a simple screening test with nasal nitric oxide (NO) a seemingly good candidate as it forms part of the European consensus guidelines [14]. However, specificity is unacceptably low in young children, precisely the group that screening needs to target. At the moment, we are therefore dependent on increased awareness of PCD by neonatologists and family doctors who should refer to specialist diagnostic centres when concerned [13]. As more genes are recognized and the cost of genetic testing comes down, it is likely that patients with even mild neonatal symptoms will be able to have DNA samples sent for screening with less need to travel to a specialist centre for brushing biopsy.

Screening and genetic testing does not replace formal diagnostic studies; sweat testing still forms part of the CF diagnostic pathway and it is likely that functional ciliary assessment will continue to be required for a diagnosis of PCD to be made. However, CF patients have benefited from increasing genotype-phenotype correlation and, in the most striking example, genotype specific treatment with ivacaftor for *G551D* mutations [56]. Greater genotype-phenotype classification is an area where PCD patients may derive most benefit from genetic testing.

## 6. Conclusions

The combination of low incidence and relatively low sensitivity of genetic testing in PCD means that general population screening is not likely to be viable in the near future, however it may be appropriate in populations where PCD is common, for example the Asian population of Bradford [3]. The incidence of situs inversus in the general population is thought to be around 1 in 10,000 with many of these cases associated with PCD [11], therefore all cases with any respiratory symptoms should be referred for assessment and the availability of rapid genetic testing may aid diagnosis. It may also be possible to screen neonates with persistent tachypnoea and other features suggestive of PCD with rapid genetic sequencing; though it would be important to consider the sensitivity of genotyping so the correct children could also be assessed using HSV and EM assessments.

PCD and CF are both autosomal recessive disorders causing chronic suppurative lung disease, raising the possibility of applying some of the CF genetic screening successes to PCD. The difficulties in achieving this reflect that CF is related to just one gene (*CFTR*) with mutation detection possible in up to 98.7% of cases whilst PCD involves 27 known and several hundred potential genes, a variety of mutations within each of these genes and, at present, the ability to detect a mutation in only 60% of cases. Additionally, in CF IRT provides a good newborn screening test, but in PCD low nNO, which is a good screening test in older children and adults, is not sufficiently specific in infancy. Genetic testing does not currently form part of the European diagnostic pathway in PCD [14], however, next generation sequencing techniques will both expand the known disease loci in PCD and improve the feasibility of rapid gene sequencing; thus increasing the role of genetic testing in PCD. Additionally, as genotype-phenotype correlation is improved, patients may benefit from more specific information on disease characteristics and, potentially, mutation specific treatments. The need for cilial structure and beat analysis is only likely to be reduced once NO screening, genetic testing sensitivity and genotype-phenotype correlation are suitably robust.
